# Shiraz medical students’ perceptions of their colleagues’ professional behavior

**Published:** 2015-07

**Authors:** MEHRDAD ASKARIAN, MOHAMMAD JAVAD EBRAHIMI NIA, FATEMEH SADEGHIPUR, MINA DANAEI, MOHSEN MOMENI

**Affiliations:** 1 Department of Community Medicine, Medicinal and Natural Products Chemistry Research Center, Shiraz University of Medical Sciences, Shiraz, Iran;; 2 Student Research Committee, Shiraz University of Medical Sciences, Shiraz, Iran;; 3 Department of Community Medicine, Shiraz University of Medical Sciences, Shiraz, Iran;; 4 Medical Informatics Research Center, Institute for Futures studies in Health, Kerman University of Medical Sciences, Kerman, Iran;; 5 Social Determinants of Health Research Center, Institute for Futures Studies in Health, Kerman University of Medical Sciences, Kerman, Iran

**Keywords:** Professionalism, Excellence, Respect, Medical students

## Abstract

**Introduction:**

Today, development of professionalism is a critical aim of medical schools. Studies have demonstrated that medical students’ perceived level of professionalism is inadequate worldwide. This study aimed to investigate the medical students’ perceptions of their colleagues’ professional behavior.

**Methods:**

This study is a cross-sectional study with 280 medical students at Shiraz University of Medical Sciences in their fifth to seventh year of study as the sample. The study was performed during one month in 2013, using stratified random sampling method. The instrument of the study was the Persian version of the questionnaire of the American Board of Internal Medicine (ABIM).The questionnaire includes demographic information, questions about the meaning of the professionalism, history of medical ethics education programs and 12 behavioral questions. The data were analyzed using student t-test and Pearson correlation test. The significance level was set as 0.05.

**Results:**

Forty percent of respondents did not know the meaning of professionalism. The mean±SD score of behavioral questions was 5.91±1.2 on a scale from 0 to 10. The mean±SD score of excellence questions was 4.94±1.7. It was 7.05±1.9 for ‘honor/integrity’, and 6.07±2.1 for ‘altruism/respect’ questions. There was a significant association between gender and excellence score (p=0.007).

**Conclusion:**

Medical students assessed their colleagues’ professional behavior as poor. They did not have proper information about professionalism. Medical students are future general practitioners and respecting medical ethics by them is very important in a perfect health system. Universities should emphasize the importance of teaching professionalism to medical students and faculty members, using innovative education methods.

## Introduction

Modern medicine has experienced new challenges including inequity in health and financing, poor adherence of professionalism by physicians and lack of positive communication between patients and doctors. It could have a negative impact on respect for the doctors (1). Recently, medical professionalism has gained special attention and development of professionalism in medical students is a critical aim of medical schools (2, 3). The concept of medical professionalism is complex but it has indicated that the priority of the patient’s interest is above the physicians’ self-interest (4).Compliance with professionalism creates trust, confidence and satisfaction in both patients and physicians (2). The American Board of Internal Medicine (ABIM) described various aspects of professionalism including altruism, accountability, excellence, honor and integrity, duty and respect for others (4).

Studies have shown that medical students with unprofessional behavior are more likely to have unprofessional practice after graduation (5). Good doctor-patient relationship is an important factor in the treatment process of patients. Therefore, medical students should participate in active learning of medical professionalism to acquire the life-long skills in this aspect of science (6). Medical students should know the definition of professionalism, its history and its application (7).In the past years, Iranian medical students learned about professionalism implicitly and informally (8).

Today in both developing and developed countries, professionalism is one of the main topics of education in medical schools. For example, in medical schools of Iran and Taiwan, attention to professionalism has recently been increased (9, 10).

Studies conducted at the university level during the last decade demonstrate the inadequacy of perceived level of professionalism among medical students or residents throughout the world. These results emphasized the importance of development of professionalism curriculum in medical schools in the future (4, 10-12). A qualitative study on Iranian medical students demonstrated their negative view about the current professionalism curriculum and teaching process (8).

Chen et al. demonstrated that clinical physicians were not satisfied with their training in medical ethics and there were some “Underperforming doctors” in this field(12). Self-assessment of professionalism among clinical faculties of Shiraz University of Medical Sciences showed that 74.9% of them reported their professionalism ability as high level. Clinical faculty teachers are a special role model for medical students. They have permanent effect on medical students’ behavior (13). Hur pointed out that there was a deep gap between professors and medical students in the perception of students' professionalism level (6).

Despite the importance of medical professionalism in medical students, to the best of our knowledge, there has not been any research about the medical students’ professional behavior in Shiraz, Iran. Shiraz University of Medical Sciences is the top university in the south of Iran. Approximately 70-100 students are admitted in Shiraz Medical School each year. To this end, this study aimed to investigate the medical students’ perceptions of their colleagues’ professional behavior. Also, we tried to evaluate the influence of demographic characteristic of the participants on their perception. 

## Methods

This cross-sectional study was conducted on the medical students being in the fifth, sixth and seventh year of study at Shiraz University of Medical Sciences. According to sample size estimation (using Cochran formula, finite population correction and withdrawal correction, considering P: 0.5, d: 0.05, CI: 95%, W: 0.2), 280 participants were selected. Using stratified random sampling method, 183participants who were doing their externships and 97ones who were doing their internships participated. The study was performed during one month in 2013. The rotation period in each ward was one month; therefore, all of questionnaires were distributed during one month to avoid bias in selection. Incompletely filled questionnaires were excluded from the survey.

The Persian version of the questionnaire of the American Board of Internal Medicine (ABIM) was used for measuring professionalism in participants (10). This questionnaire contains demographic information including age, gender, marital status, and education grade. There is 1 question about the meaning of the professionalism, 2questions about the history of passing a medical ethics course and 1 question about the personal study in the field of medical ethics. Each of these questions has a yes (correct) or a no (incorrect) answer.

There were 12 questions regarding the respondents’ perceptions about their colleagues’ professional behavior. These questions were divided in to 3 subtitle including excellence, honor/integrity, and altruism/respect, respectively. The content validity of the Persian version of this questionnaire was assessed by expert opinions, especially the opinions of  Aramesh that used this questionnaire in one survey measuring professionalism in residents of Tehran University (10).The reliability of this questionnaire was checked. The Total Cronbach's alpha for all questions on respondents’ perceptions about their colleagues’ professional behavior was 0.73, being 0.87, 0.75, and 0.73 for Excellence Questions, Honor/integrity Questions and Altruism/respect Questions, respectively.

The first five behavioral questions measured the participants’ perception of correct performance of colleagues in professionalism and others measured their incorrect performance in this field. For score homogenization, reverse scoring was used for the answers of questions 6 to 12. The numerical scoring scale (score of 0-10) was run in the opposite direction (score of 10-0).Therefore in all behavioral questions, 10 demonstrated the highest level of medical professionalism and 0 represented the lowest. Finally, to measure how precise one answered the questions, respondents estimated their precision form 1 to 5 scale.

After obtaining a permit and letter of introduction from the Research Council of Shiraz University of Medical Sciences, the investigators visited the University Hospitals (Namazi, Shahid Faghihi, Hafez, Shahid Chamran, and Shahid Dastgheib) in their daily working hours. The self-reported questionnaires were distributed between the participants, using convenient sampling method. Written consent was obtained by mentioning the elements of the consent at the top of the questionnaire.

The data were analyzed using SPSS 14. Standard descriptive statistics were reported. The statistical differences in the mean scores were compared between various subgroups, using student t-test and Pearson correlation test. The significance level was set as 0.05. 

## Results

**Table1:** The participants’ answers to the meaning of professionalism and studying medical ethics

**Questions**	**Participants answers** **N (%)**	
	**No**	**Yes**
Knowing the meaning of the “Professionalism”	112 (41.9)	155 (58.1)
Passing the medical ethics course	44 (15.9)	233 (84.1)
Passing other educational courses related to the medical ethics	237 (85.3)	41 (14.7)
Having a personal study in the field of medical ethics	226 (81)	53 (19)

The frequency of participants’ answers to the behavioral questions is shown in table 2.

**Table 2:** The participants’ answers to the behavioral questions

**Behavioral questions**	**Participants answers N (%)**			
	**0 scale (Never)**	**1-5 scale**	**6-9 scale**	**10 scale (Always)**
Excellence items				
**Having individuals as role models**	6 (2.2)	114 (41)	139 (50)	19 (6.8)
**Encountering individuals who display and promote professional behavior**	3 (1.1)	120 (43.2)	139 (50)	16 (5.7)
**Attaining educational materials for patients**	39 (14.1)	176 (63.8)	59 (21.4)	2 (0.7)
**Placing the needs of patients ahead of self-interest**	7 (2.5)	184 (66.2)	86 (30.9)	1 (0.4)
**Educating patients about their illnesses**	5 (1.7)	157 (56.5)	115 (41.4)	1 (0.4)
Honor/integrity questions				
**Holding data from a patient’s chart without being given an explanation from resident or attending physician**	76 (27.5)	149 (54)	49 (17.8)	2 (0.7)
**Lying to a patient**	60 (21.7)	183 (66.1)	33 (11.9)	1 (0.3)
**Asking colleagues to write orders or fill out forms **	35 (12.8)	105 (38.5)	121 (44.3)	12 (4.4)
** Asking colleagues to copy their history and physical examination**	96(34.7)	146 (52.7)	33 (11.9)	2 (0.7)
Altruism/respect questions				
**Referring to patients as ‘hits, gamers, real citizens, or other terms’**	74 (26.7)	156 (56.3)	45 (16.3)	2 (0.7)
**Making derogatory statements about their colleagues or other health care workers.**	20 (7.2)	167 (60.3)	79 (28.5)	11 (4)
**scheduling tests or performing procedures at times that are more convenient for students than for the patient**	8 (2.9)	158 (56.6)	105 (37.6)	8 (2.9)

The mean±SD score of behavioral questions was 5.91±1.2 on a scale from 0 to 10. As table 3 shows there is no significant relationship between the demographic characteristics and the behavioral score. The correlation of age and behavioral questions was not significant (r=0.02, p=0.732).

**Table 3:** Comparing practice score of participants according to demographic characteristics

**Demographic characteristics**	**Total behavioral score (Mean±SD)**	**p**	**Confidence interval of the difference**
Gender			
Male	6.04±1.21	0.082	-0.03-0.55
Female	5.787±1.21		
Marital status			
Single	5.94±1.3	0.291	-0.15-0.57
Married	5.74±1.2		
Education grade			
Externship	5.95±1.25	0.520	-0.20-0.39
Internship	5.85±1.23		

The mean±SD item score of excellence questions was 4.94±1.7 out of the maximum of 10.Table 4 shows a significant association between gender and excellence score (p=0.007). The correlation of age and excellence score was not significant (r=0.04, p=0.523).

As to the aspect of honor/integrity, the mean±SD item score was 7.05±1.9 out of the maximum of 10. There was no significant association between demographic characteristics and behavioral score (Table5). The correlation of age and honor/integrity score was not significant (r=0.02, p=0.790).

The mean±SD item score of altruism/respect questions was 6.07±2.1 out of the maximum 10. As table 4 shows there is no significant association between the demographic characteristics and the altruism/respect score. The correlation of age and altruism/respect score was not significant (r= -0.02, p=0.724).

**Table 4:** Comparing excellence, honor/integrity and altruism/respect score of participants according to the demographic characteristics

**Demographic characteristics**	**Behavioral score (Mean±SD)**	**p**	**Confidence interval of the difference**
Excellence score			
Gender			
Male	5.25±1.8	0.007	0.21-0.90
Female	6.68±1.7		
Marital Status			
Single	4.98±1.8	0.298	-0.24-0.85
Married	4.71±1.7		
Education grade			
Externship	4.87±1.6	0.393	-0.61- 0.24
Internship	5.05±1.8		
Honor/integrity score			
Gender			
Male	7.02±1.9	0.893	-0.51-0.44
Female	7.05±2.0		
Marital status			
Single	7.01±2.1	0.654	-0.77-0.45
Married	7.04±1.6		
Education grade			
Externship	7.15±1.9	0.310	-0.23-0.73
Internship	6.90±2.0		
Altruism/respect score			
Gender			
Male	6.15±2.1	0.461	-0.31-0.70
Female	5.96±2.2		
Marital Status			
Single	6.14±2.1	0.063	-0.03-0.70
Married	5.61±2.2		
Education grade			
Externship	6.19±2.1	0.221	-0.19-0.83
Internship	5.87±2.1		

Approximately 3% of the participants described their accuracy in filling the questionnaire as very low or low, 27% as moderate and 70% as high or very high. 

## Discussion

This study demonstrated that the medical students assessed their colleagues’ professional behavior as poor. Medical students are future general practitioners and the observance of medical ethics by them is very important in a perfect health system. The behavioral questions mean item score was 5.91, on a scale of 0 to 10. Aramesh (9) and Deliza (4) reached the same results in medical residents (mean±SD: 6.12±0.4) and physiatrist residents (mean±SD: 7.7±1), respectively. The mean score of behavioral questions in this study is lower than that in the other two studies; this difference could be due to difference between statistical population (5 to 7 years medical students, and residents) in these studies. Residents have more responsibility, clinical experience and involvement in legal aspects of medicine than medical students. Also the formal and informal educational programs in these two populations are different (14, 15). Residents are good teachers for medical students and we can use these potential resources for practical education to medical students in hospitals.

Comparing the item scores of subscales showed that the lowest score was assigned to excellence items. Priority of the patients’ interest above physicians’ self-interest is the main concept of professionalism (4) and this was ignored by a high percentage of the medical students. The same results were reached by Deliza (4) and Aramesh (9). These findings showed that training programs should be designed to meet this aspect of the professionalism.

Role modeling is powerful and a critical teaching tool in medicine (16). Bazrafcan found that a high percentage of clinical faculties of Shiraz University of Medical Sciences reported their professionalism ability as high level (13) but medical students’ opinion showed the opposite because this item gaineda low score by many students.  The clinical faculty members should be familiar with the principles of medical ethics and its applications because they are role models for their students (17). Therefore, providing education programs for clinical faculty members could help the improvement of quality of medical ethics in students. According to the importance of role modeling, self-monitoring and peer monitoring of professionalism ability by clinical faculties is necessary.

Despite passing the ethics course, many participants did not know the meaning of professionalism. Medical students do not understand the importance of medical ethics; therefore, they do not pay enough attention to the ethics course as they do to the other courses. Also they are not capable of using ethical theory in practice.Swick (18) and Hafferty (19) had the same results. The strategies that are used to teach professionalism are inadequate. Universities should offer the medical ethics and professionalism courses to medical students, using the single perfect strategy for all medical schools to ensure the technical accuracy of training process. Also medical students who are doing their externships and internships need to pass the practical courses of professionalism to obtain a deeper understanding of this concept and its importance.

There was no significant association between medical students’ perceptions of their colleagues’ professional behavior and their demographic characteristics. Male participants scored higher than their female colleagues in the ‘excellence’ item. Other studies find a significant association between gender and professionalism(20, 21). This finding emphasized that audience analysis is fundamental to the success of medical students’ educational programs.

More than 70 percent of the participants mentioned that their accuracy in filling the questionnaire was high or very high. This implies the accuracy and reliability of the results of this study. On the other hand, this study was based on self-administered questionnaires and we measured medical students’ perceptions of their colleagues’ professional behavior instead of direct measurement of students’ behavior. Further direct observational studies and interventional studies for assessing the impact of training on medical students’ adherence to medical ethics are required. 

## Conclusion

Medical students do not have proper information about professionalism. They have not positive perception on the observance of medical ethics by their colleagues. Universities should emphasize the importance of teaching professionalism to medical students and faculty members based on audience analysis of educational methods, using innovative educational methods. 
